# Liposomes for Use in Gene Delivery

**DOI:** 10.1155/2011/326497

**Published:** 2010-12-15

**Authors:** Daniel A. Balazs, WT. Godbey

**Affiliations:** Laboratory for Gene Therapy and Cellular Engineering, Department of Chemical and Biomolecular Engineering, Tulane University, 6823 St. Charles Avenue, 300 Lindy Boggs Center, New Orleans, LA 70118, USA

## Abstract

Liposomes have a wide array of uses that have been continuously expanded and improved upon since first being observed to self-assemble into vesicular structures. These arrangements can be found in many shapes and sizes depending on lipid composition. Liposomes are often used to deliver a molecular cargo such as DNA for therapeutic benefit. The lipids used to form such lipoplexes can be cationic, anionic, neutral, or a mixture thereof. Herein physical packing parameters and specific lipids used for gene delivery will be discussed, with lipids classified according to overall charge.

## 1. Introduction

Liposomes are vesicular structures that can form via the accumulation of lipids interacting with one another in an energetically favorable manner. Depending upon the structure and the composition of the bulk solution, liposomes can separate hydrophobic or hydrophilic molecules from the solution. These vesicles are not rigid formations but rather are fluid entities that are versatile supramolecular assemblies. Because they have dynamic properties and are relatively easy to manipulate, liposomes have been used widely in the analytical sciences as well as for drug and gene delivery. Since their first published use in 1965 [1, 2], the value and practicality of liposome functions have been recognized and continually improved upon. 

The advances that brought about liposome-derived technologies have been recognized as some of the cornerstones of bionanotechnology [3]. The unique advantages imparted by lipid vesicles are their diverse range of morphologies, compositions, abilities to envelope and protect many types of therapeutic biomolecules, lack of immunogenic response, low cost, and their differential release characteristics [4–6]. These characteristics have led to applications in chemical and biochemical analytics, cosmetics, food technologies, and drug and gene delivery [7, 8]. There are numerous lipid formulations for each of these applications. However, this review will focus primarily on the use of liposomes for gene delivery.

## 2. Characteristics

**Figure 1:**
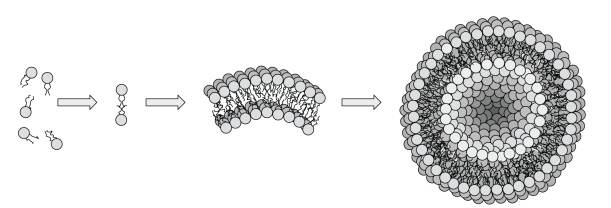
Certain amphipathic lipid molecules in aqueous solution spontaneously form leaflets, then bilayer membranes, and eventually liposomes.

Liposomes are generally formed by the self-assembly of dissolved lipid molecules, each of which contains a hydrophilic head group and hydrophobic tails. These lipids take on associations which yield entropically favorable states of low free energy, in some cases forming bimolecular lipid leaflets (Figure 1). Such leaflets are characterized by hydrophobic hydrocarbon tails facing each other and hydrophilic head groups facing outward to associate with aqueous solution [9]. At this point, the bilayer formation is still energetically unfavorable because the hydrophobic parts of the molecules are still in contact with water, a problem that is overcome through curvature of the forming bilayer membrane upon itself to form a vesicle with closed edges [10] (Figure 1). This free-energy-driven self-assembly is stable and has been exploited as a powerful mechanism for engineering liposomes specifically to the needs of a given system [11].

Lipid molecules used in liposomes are conserved entities with a head group and hydrophobic hydrocarbon tails connected via a backbone linker such as glycerol [12]. Cationic lipids commonly attain a positive charge through one or more amines present in the polar head group. The presence of positively charged amines facilitates binding with anions such as those found in DNA. The liposome thus formed is a result of energetic contributions by Van der Waals forces and electrostatic binding to the DNA which partially dictates liposome shapes [13]. Because of the polyanionic nature of DNA, cationic (and neutral) lipids are typically used for gene delivery, while the use of anionic liposomes has been fairly restricted to the delivery of other therapeutic macromolecules [14].

Liposomes can exhibit a range of sizes and morphologies upon the assembly of pure lipids or lipid mixtures suspended in an aqueous medium [2]. A common morphology which is analogous to the eukaryotic cellular membrane is the unilamellar vesicle. This vesicle is characterized by a single bilayer membrane which encapsulates an internal aqueous solution, thus separating it from the external (bulk) solution [15]. Both cationic amine head groups and anionic phospholipid head groups can form these single-walled vesicles. Vesicle sizes fall into the nanometer to micrometer range: small unilamellar vesicles are 20–200 nm, large unilamellar vesicles are 200 nm–1 *μ*m, and giant unilamellar vesicles are larger than 1 *μ*m [2]. 

Giant vesicles also include other morphologies such as multilamellar, which consists of multiple concentric bilayers, oligolamellar, which consists of only two concentric bilayers, and multivesicular, which consists of multiple smaller unilamellar vesicles inside of one giant one. With the exception of multilamellar vesicles, these other morphologies are difficult to obtain without highly controlled processes for formation [2]. Giant vesicles also deserve special attention because their sizes are large, ranging from 1 *μ*m to more than 100 *μ*m [2]. These large vesicles are studied and well characterized, partially due to the ease of observation via optical microscopy [10].

During the compaction of polynucleotides into liposomal assemblies, a number of structures have been known to appear [5, 6, 16–19]. Each structure is formed in the most energetically favorable conformation based upon characteristics of the specific lipids used in the system [13]. A dependent term known as the structure-packing parameter can be used to suggest what shape the amphiphile will take, depending on the ratio of size variables. The packing parameter is defined as

(1) $$P=\frac{v}{al_{c}},$$

where *v*: the volume of the hydrocarbon portion, *a*: the effective area of the head group, and *l*_*c*_: the length of the lipid tail. 

**Figure 2:**
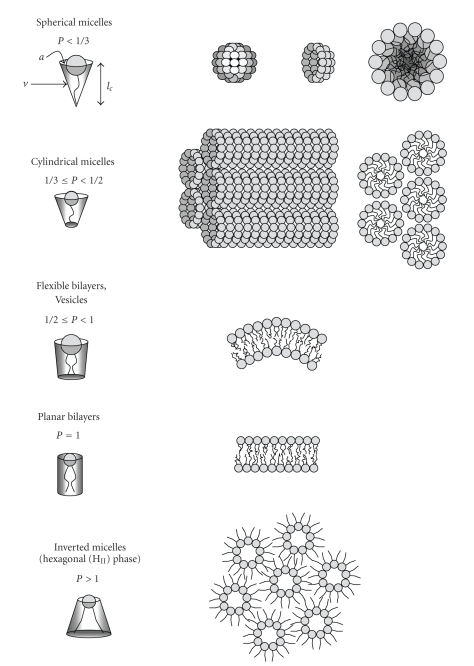
Structures predicted by the packing parameter P.

This correlation predicts a range of structures according to the following conditions [13, 20] (Figure 2):

(2) $$\begin{array}{cc} & P<\frac{1}{3}\to \text{spherical}\;\text{micelle},\\ & \frac{1}{3}\leq P<\;\frac{1}{2}\to \text{cylindrical}\;\text{micelle},\\ & \frac{1}{2}\leq P<1\to \text{flexible}\;\text{bilayers},\text{vesicles},\\ & P=1\to \text{planar}\;\text{bilayers},\\ & P>1\to \text{inverted}\;\text{micelles},\left(\text{hexagonal}\;\left({\text{H}}_{\text{II}}\right)\text{phase}\right). \end{array}$$

## 3. Cationic Lipids

A solution of cationic lipids, often formed with neutral helper lipids, can be mixed with DNA to form a positively charged complex termed a lipoplex [21]. Well-characterized and widely used commercial reagents for cationic lipid transfection include N-[1-(2,3-dioleyloxy)propyl]-N,N,N-trimethylammonium chloride(DOTMA) [22], [1,2-bis(oleoyloxy)-3-(trimethylammonio)propane] (DOTAP) [23], 3*β*[N-(N′, N′-dimethylaminoethane)-carbamoyl] cholesterol (DC-Chol) [24], and dioctadecylamidoglycylspermine (DOGS) [25]. Dioleoylphosphatidylethanolamine (DOPE), a neutral lipid, is often used in conjunction with cationic lipids because of its membrane destabilizing effects at low pH, which aide in endolysosomal escape [26].

Many cationic lipid compounds have been formulated since the advent of DOTMA [27–31]. Each lipid has different structural aspects, such as head group size and hydrocarbon tail length. These aspects confer distinct characteristics to the lipid/DNA complex, which in turn affect association with and uptake into the cell. However, the basic structure of cationic lipids mimics the chemical and physical attributes of biological lipids [32]. The positive charge on the head group facilitates spontaneous electrostatic interaction with DNA, as well as binding of the resulting lipoplexes to the negatively charged components of the cell membrane prior to cellular uptake [33, 34]. The use of a cation is a recurring theme for virtually all chemically mediated gene delivery vectors, including polymers, lipids, and nondegradable nanoparticles. 

Between 8–18 carbons commonly comprise the hydrocarbon tails of lipids used for gene delivery. The tails are typically saturated, but a single double bond is occasionally seen. The combination of hydrocarbon chains in a lipid mixture can be symmetric or asymmetric. It has been shown that certain asymmetric lipid mixtures with both shorter saturated carbon chains and long unsaturated carbon chains produce relatively high transfection efficiencies as compared to mixed formulations of symmetric cationic lipids [35]. 

Hydrophobic tails are not the only liposomal features that play a role in effective gene delivery—ionizable head groups are also involved. Some examples are the multivalent cationic lipids DOSPA and DOGS (covered in Section 3.2); both of which have a functionalized spermine head group that confers the ability to act as a buffer, such as in the case where there is an influx of protons into a maturing endosome/endolysosome [36]. Such buffering could extend the amount of time needed to activate acid hydrolases and could explain why some multivalent cationic lipids can exhibit higher transfection efficiencies versus their monovalent counterparts [25, 37]. 

### 3.1. Monovalent Cationic Lipids

#### 3.1.1. DOTMA (see Figure 3)

N-[1-(2,3-dioleyloxy) propyl]-N,N,N-trimethylammonium chloride, or DOTMA, was one of the first synthesized and commercially available cationic lipids used for gene delivery. Its structure consists of 2 unsaturated oleoyl chains (C18 : Δ^9^), bound by an ether bond to the three-carbon skeleton of a glycerol, with a quaternary amine as the cationic head group [22]. As compared to other methods of gene transfer used in the late 1980s, DOTMA proved to facilitate up to 100-fold more efficient gene delivery than the use of DEAE-dextran coprecipitation or calcium phosphate [22]. The ability to entrap DNA or RNA in a liposome in a relatively simple fashion, with effective gene delivery to cells, significantly influenced and improved the potential of nonviral agents for gene therapy [22, 38]. Based upon the use of comparative protein expression assays such as luciferase, *β*-galactosidase, or chloramphenicol acetyltransferase, initial success of *in vitro* transfection of multiple cell lines with DOTMA sparked a number of attempts to improve the lipid formulation and resulted in the creation of many effective formulations including such notable lipids as DOTAP [23] (see Section 3.1.2) and DC-Chol [24] (Section 3.1.3).

Commercialization of DOTMA as Lipofectin involved its coupling with DOPE (Section 4.1) in a 1/1 ratio due to the ability of DOPE to increase transfection efficiencies. Once commercialized, improvements in Lipofectin were desired, motivating others to add functional groups to the DOTMA. Many alterations made in the four major moieties of DOTMA (head group, linker, linkage bonds, and hydrocarbon chains) have reflected widespread efforts to reduce toxicity and increase transfection efficiencies [23, 39]. These studies have suggested, however, that cytotoxicities associated with the formulated monovalent lipids were dependent on plated cell density. Plate densities of 25%–35%, treated with cationic lipoplexes, yielded roughly half the amount of cell protein per plate versus controls. Near-confluent cell monolayers exhibited very little evidence of cytotoxicity. These findings supported a need for manipulations in the structural aspects of the lipids for lowered cytotoxicity in subconfluent populations [23]. Felgner et al. [40] also experimented with novel lipid formulations by altering DOTMA to obtain a more robust understanding of the mechanism of biological action. The structural changes included different combinations of side chains and alkyl attachments to the head groups, as well as the replacement of a methyl group on the quaternary amine of DOTMA with a hydroxyl. Their report suggested that compounds with such a hydroxyl modification display improved protein expression after transfection by two- to three-fold over those observed following DOTMA-mediated transfections. Stabilization of the bilayer vesicles was purported to occur as a result of the hydroxyl group remaining in contact with the aqueous layer surrounding the liposome. Compounds lacking this moiety were hypothesized to become entrenched in the aliphatic region, thus destabilizing the membrane. It was also indicated that aliphatic chain length had a large effect on the efficacy of lipid vectors. As the lengths of the saturated chains were increased in the DOTMA analogs, transfection efficiencies decreased. This was thought to be due to increased bilayer stiffness, which may have prevented efficient fluid interactions with the endosomal membrane to thus hamper the release of the liposomes or plasmid DNA from the endosomal compartments.

#### 3.1.2. DOTAP (see Figure 4)

[1,2-bis(oleoyloxy)-3-(trimethylammonio)propane], or DOTAP, was first synthesized by Leventis and Silvius in 1990 [23]. The molecule consists of a quaternary amine head group coupled to a glycerol backbone with two oleoyl chains. The only differences between this molecule and DOTMA are that ester bonds link the chains to the backbone rather than ether bonds. It was originally hypothesized that ester bonds, which are hydrolysable, could render the lipid biodegradable and reduce cytotoxicity. This study showed that the transfection activities and levels of cytotoxicity associated with DOTAP/DOPE formulations are not statistically different from those associated with DOTMA/DOPE composites. Notably, this type of monovalent lipids also showed little to no cytotoxic effect on near-confluent cell monolayers, in addition to exhibiting the same lipoplex sensitivity at 25%–35% cell confluence as mentioned in Section 3.1.1 [23].

The use of 100% DOTAP for gene delivery is inefficient due to the density of positive charges on the liposome surface, which possibly prevents counter ion exchange [41]. DOTAP is completely protonated at pH 7.4 (which is not the case for all other cationic lipids) [41], so it is possible that more energy is required to separate the DNA from the lipoplex for successful transfection [42]. Thus, for DOTAP to be more effective in gene delivery, it should be combined with a helper lipid, as seems to be the case for most cationic lipid formulations.

High temperature and long incubation times have been used to create lipoplexes that exhibit resistance to serum interaction [43]. Interestingly, this approach was only observed to affect monovalent cationic lipids such as DOTMA, DOTAP, or DC-Chol, as opposed to multivalent cationic lipids. The specific reasons for this phenomenon remain unclear. In fact, the specific mechanism behind serum inactivation of lipoplexes in general is as yet unexplained. Several hypotheses have been offered as to the mechanism, including the prevention of lipoplex binding to cell membranes by serum proteins [34, 43], the prevention of structural complex maturation by serum proteins binding to cationic charges on the lipoplexes [43], and the disparity of endocytosis pathways—which have varying kinetics—that are used for lipoplex endocytosis, with the method of endocytosis being regulated by the size of the lipoplexes or aggregates of lipoplexes plus serum proteins [34, 44].

#### 3.1.3. DC-Chol (see Figure 5)

3*β*[N-(N',N'-dimethylaminoethane)-carbamoyl]cholesterol, or DC-Chol, was first synthesized by Gao and Huang in 1991 [24]. DC-Chol contains a cholesterol moiety attached by an ester bond to a hydrolysable dimethylethylenediamine. Cholesterol was reportedly chosen for its biocompatibility and the stability it imparts to lipid membranes, an idea which was supported by observed transfection activity of up to two- to four-fold greater chloramphenicol acetyltransferase expression (CAT assay). Additionally, DC-Chol was found to have a four-fold reduction in cytotoxicity versus Lipofectin in some cell lines [24].

In contrast to cationic liposomes containing fully charged quaternary amines (e.g. DOTMA and DOTAP), DC-Chol, in a 1 : 1 lipid ratio with DOPE, contains a tertiary amine that is charged on 50% of the liposome surface at pH 7.4 [45]. This feature is thought to reduce the aggregation of lipoplexes leading to higher transgene expression [46]. The reduction in overall lipoplex charge can also aid in DNA dissociation during gene delivery [41], which has been proven to be necessary for successful transfection [42]. 

### 3.2. Multivalent Cationic Lipids

#### 3.2.1. DOSPA (see Figure 6)

{2,3-dioleyloxy-N-[2(sperminecarboxamido)ethyl]-N,N-dimethyl-l-propanaminium trifluoroacetate}, or DOSPA, is another cationic lipid synthesized as a derivative of DOTMA. The structure is similar to DOTMA except for a spermine group which is bound via a peptide bond to the hydrophobic chains. This cationic lipid, used with the neutral helper lipid DOPE at a 3 : 1 ratio, is commercially available as the transfection reagent Lipofectamine. In general, the addition of the spermine functional group allows for a more efficient packing of DNA in terms of liposome size. The efficient condensation is possibly due to the many ammonium groups in spermine. It has been shown that spermine can interact via hydrogen bonds with the bases of DNA in such a way as to be attracted on one strand and wind around the major groove to interact with complementary bases of the opposite strand [47].

#### 3.2.2. DOGS (see Figure 7)

Di-octadecyl-amido-glycyl-spermine, or DOGS, has a structure similar to DOSPA; both molecules have a multivalent spermine head group and two 18-carbon alkyl chains. However, the chains in DOGS are saturated, are linked to the head group through a peptide bond, and lack a quaternary amine. DOGS is commercially available under the name Transfectam. This lipid has been used to transfect many cell lines, with transgene expression levels more than 10-fold greater than those seen following calcium phosphate transfections [25]. In addition, Behr et al. showed that not only was DOGS very effective in delivering the CAT reporter plasmid, but it was also associated with no noticeable cytotoxicity [25].

Much like the multivalent cationic lipid DOSPA, DOGS is very efficient at binding and packing DNA, a result of the spermine head group that so closely associates with DNA [25]. Characterization of the head group of DOGS was determined to facilitate not only efficient condensation of DNA but also buffering of the endosomal compartment, which was thought to protect the delivered DNA from degradation by pH-sensitive nucleases [36]. DOGS is a multifaceted molecule in terms of buffering capacity. At pH values lower than 4.6 all of the amino groups in the spermine are protonated, while at pH = 8 only two are purportedly ionized, which promotes arrangement into a lamellar structure [48]. The packing ability of DOGS is due, in part, to the dynamics of the large head group molecule and the length of long unsaturated carbon chains.

### 3.3. Modifications for Improved Liposome-Mediated Gene Delivery

#### 3.3.1. Poly(ethylene) Glycol

Recent improvements in lipofection have facilitated protection from degradation *in vivo*, due to surface modifications with polyethylene glycol (PEG). PEG presents many attractive qualities as a liposomal coating, such as availability in a variety of molecular weights, lack of toxicity, ready excretion by the kidneys, and ease of application [49]. Methods of modifying liposomal surfaces with PEG include its physical adsorption onto the liposomal surface and its covalent attachment onto premade liposomes [50]. 

It has been shown by Kim et al. [51] that PEGylated lipoplexes yield increased transfection efficiencies in the presence of serum as compared to liposomal transfection methods lacking such surface attachments. Additionally, the PEGylated lipoplexes display improved stabilities and longer circulation times in the blood. It is thought that the PEG forms a steric barrier around the lipoplexes, which stifles clearance due to reduced macrophage uptake [50], and may allow the liposome to overcome aggregation problems through mutually repulsive interactions between the PEG molecules [52]. These characteristics increase bioavailability, facilitating higher transfection efficiencies due to improved tissue distribution and larger available concentrations [53]. 

Because of the decreased immune responses and increased circulation times associated with PEG-modified liposomes, these particles are sometimes referred to as “stealth liposomes.” However, such liposomes lack specificity with regard to cellular targeting. Notably, Shi et al. found that PEGylation inhibited endocytosis of the lipoplexes in a fashion that was dependent upon the mole percentage of PEG on the liposome, as well as the identity of certain functional groups that were conjugated to the lipoplexes [54]. Additionally, upon incorporation into the cell, PEG worked to deter proper complex dissociation by stabilizing a lamellar phase of DNA packing. As a result of these findings, a need has arisen for the creation of novel PEG-containing liposomes whereby the attached PEG is removed following endocytosis via a hydrolysable connecting molecule.

#### 3.3.2. Additions and Alternatives to Poly(ethylene) Glycol

Alternative liposomal formulations utilizing polymers other than PEG are being produced with the goal of creating sterically protected lipoplexes. Additional aims of such systems include biocompatibility, flexible structure, and solubility in physiological systems [50]. A report by Metselaar et al. on the use of L-amino-acid-based polymers for lipoplex modification found an extended circulation time and reduced clearance by macrophages at levels similar to those seen with lipoplexes modified with PEG. Results suggested that approximately 10% of the injected dose of the L-amino-acid-modified complexes was still present in the blood of treated rats after 48 hours [49]. These oligopeptides are attractive alternatives to PEG due to advantages such as increased biodegradability and favorable pharmacokinetics when lower concentrations are used per dose.

Liposomes can also be coupled to targeting moieties through the use of PEG to impart attraction to affected tissues for optimal routing and transfection. Targeting ligands are selected based upon specific target cell receptors. The target cells can be normal or transformed (tumor) cells. Examples of such ligands include transferrin [55], a popular ligand for delivery of anticancer drugs to solid tumors *in vivo*, and haloperidol [56], a ligand that associates with sigma receptors that are overexpressed in many types of cancer.

## 4. Neutral Lipids

### 4.1. DOPE and DOPC (see Figure 8)

Most liposomal formulations used for gene delivery consist of a combination of charged lipids and neutral helper lipids [12, 22–24, 26, 28]. The neutral helper lipids used are often dioleoylphosphatidylethanolamine (DOPE), which is the most widely used neutral helper lipid, or dioleoylphosphatidylcholine (DOPC). Results have shown that the use of DOPE versus DOPC as the helper lipid yields higher transfection efficiencies in many cell types [28, 57], thought to be due to a conformational shift to an inverted hexagonal packing structure (Figure 2) that is imparted by DOPE at low pH. In contrast to the creation of repeated layers of DNA/lipids, as is the case in lamellar packing, the inverted hexagonal packing structure is similar to that of a honeycomb of tubular structures which condense DNA inside the tubes through electrostatic interactions. The tubes aggregate due to Van der Waals interactions between the lipid tails that spread out to encircle each tube. Fusion and destabilization of the lipoplexes during transfection are thought to occur due to the exposure of the endosomal membrane to invasive hydrocarbon chains [58]. Studies have suggested that a hexagonal conformation allows for efficient escape of complexed DNA from endosomal vesicles via destabilization of the vesicle membrane [17, 59]. With the lysosomotropic agent chloroquine inhibiting the activity of DOPE-containing lipoplexes, it is reasonable to assume that the membrane-destabilizing hexagonal conformation associated with DOPE is brought about at acidic pH [26]. 

In DOTAP-mediated DNA-binding studies, it was discovered that liposomes—formulated without DOPE—would not effectively complex with DNA to neutralize it until a 2 : 1 N : P ratio was reached due to an inability to displace counter ions bound to the cationic lipid head groups [41]. In contrast, complexes with a 1 : 1 ratio of DOTAP/DOPE continuously neutralized and complexed with the negatively charged DNA at all charge ratios. This is possibly due to salt bridges more easily forming between the positively charged head groups of the cationic lipids and the phosphate groups of DOPE moieties. This association would force the primary amine of DOPE to stabilize itself in the plane of the liposome surface and allow for more close interactions with the negatively charged phosphate of the DNA. DOPE could also facilitate counter ion release from the positively charged lipid head group, thus lowering the energy required for binding DNA [41]. Circular dichroism has been used to indicate that the use of DOPE as a helper lipid allows for much closer contact and packing of DNA helices [41]. 

DC-Chol and other cholesterol derivatives have been incorporated into lipoplex assembly for increased transfection efficiency *in vivo* [60, 61]. Galactosylated cholesterol derivatives have been shown to lower cytotoxicity levels and improve transfection efficiencies in human hepatoma cells (Hep G2), likely due to the affinity of cellular receptors for galactosylated ligands [62]. This result indicates that lipoplexes can be formulated for cell-specific uptake through the addition of specific ligands. 

## 5. Anionic Lipids

**Figure 9:**
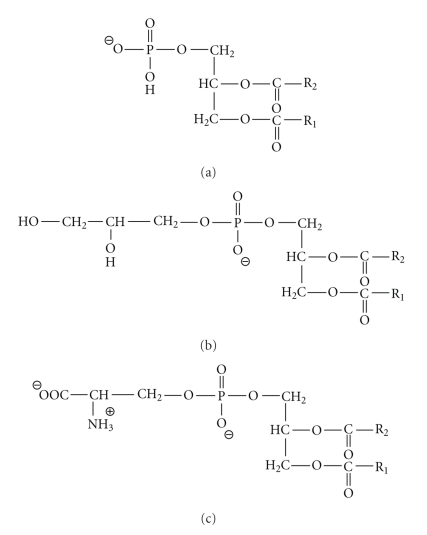
Anionic Lipids. (a) Phosphatidic acid (pH = 7). (b) Phosphatidylglycerol. (c) Phosphatidylserine.

In general, gene delivery by anionic lipids is not very efficient. The negatively charged head group prevents efficient DNA compaction due to repulsive electrostatic forces that occur between the phosphate backbone of DNA and the anionic head groups of the lipids. However, due to the fact that cationic liposomes can be inactivated in the presence of serum, are unstable upon storage, and exhibit some cytotoxicity both *in vitro* and *in vivo*, anionic liposomes have been studied as potential gene delivery vehicles [63–65]. Formation of DNA-containing liposomes using anionic lipids can be brought about through the use of divalent cations to negate the mutual electrostatic repulsion and facilitate lipoplex assembly [8]. Anionic lipoplexes are composed of physiologically safe components including anionic lipids, cations, and plasmid DNA [66]. Commonly used lipids in this category are phospholipids that can be found naturally in cellular membranes such as phosphatidic acid, phosphatidylglycerol, and phosphatidylserine (Figure 9). As with the lipids presented earlier, anionic lipids can contain any of a wide range of fatty acid chains in the hydrophobic region. The specific fatty acids incorporated are responsible for the fluidic characteristics of the liposome in terms of phase behavior and elasticity [2]. Perhaps due to the natural presence of these specific phospholipids in the host cell membrane, gene delivery via lipoplexes with net negative surface potentials has been associated with lower clearance and phagocytosis by macrophages, which is consistent with favorable biocompatibility [67]. 

Various anionic liposomes have been characterized for gene delivery in a small number of cell types including CHO cells and primary hippocampal neurons [8, 66, 68, 69]. While such investigations are novel, overall knowledge regarding anionic lipofection is as yet limited due to a lack of extensive testing; DNA entrapment in anionic liposomes is still inefficient, and cytotoxicity data remain inadequate. 

Divalent cations can be incorporated into the system to enable the condensation of nucleic acids prior to envelopment by anionic lipids. Several divalent cations have been tested for use in anionic lipoplexes such as Ca^2+^, Mg^2+^, Mn^2+^, and Ba^2+^, but it has been observed that the use of Ca^2+^ yielded the highest transfection efficiency due to its higher DNA binding affinity [70, 71]. An investigation conducted by Srinivasan and Burgess confirmed that Ca^2+^ was the most effective cation for DNA compaction as compared to Na^+^ and Mg^2+^ [66]. This affinity is potentially a result of the smaller hydrodynamic radius of calcium which gives a larger charge per unit surface area. The use of Ca^2+^ not only overcame the strong electrostatic repulsion between the DNA and the lipids, but also promoted uptake of the lipoplexes by the cell [8]. However, the use of high concentrations of calcium (in excess of 25 mM) was shown to be detrimental to transfection efficiency because of the creation of aggregate lipoplexes, having particle sizes of 500 nm and higher [66]. Optimum transfection efficiency is achieved with particles sizes of about 200 nm due to factors thought to be related to clathrin-mediated uptake [72]. 

Mixtures of the anionic lipid dioleoylphosphatidylglycerol (DOPG) and the neutral lipid DOPE have been investigated to determine an optimal ratio for transfection [66]. It was suggested that a 1 : 4 ratio of DOPG to DOPE was a proper balance to allow the negatively charged phospholipids to form lipoplexes while still having enough of the neutrally charged phospholipids to allow for endosomal escape. DOPG has a packing parameter less than 1 and tends to form flexible bilayers and vesicles (Figure 2) [73]. This characteristic can be contrasted to that of DOPE, which has a packing parameter greater than one and is known to adopt an inverted hexagonal structure that promotes membrane destabilization [13, 70]. Transmission electron microscopy revealed that this particular formulation yields liposomes of a spherical multilamellar structure [66]. However, upon relocation to the late endosome or endolysosome, the lipoplex may alter its morphology due to the effects of pH upon the DOPE. The 1 : 4 ratio was seen to exhibit higher transfection efficiency and cell viability versus the cationic formulation Lipofectamine 2000 [66]. 

Despite some favorable investigations into the use of anionic liposomes for gene delivery, there are some potential downfalls associated with systemic delivery that must be further explored. Some studies have indicated that, upon exposure to certain plasma lipoproteins, destabilization and leakage of liposomal contents can occur. For example, in liposomes lacking cholesterol, high density lipoprotein can cause some disintegration of the liposome [74]. However, in liposomes which do contain cholesterol, low density lipoproteins can also cause leakage of contents [75]. Characterization studies like these are very useful in terms of determining what mole percentages and types of lipids must be taken away or added to liposomal formulations to obtain maximum delivery of a desired cargo.

## 6. Concluding Remarks

An abundance of uses for liposomes has been investigated since their introduction into the scientific literature in the 1960s. These studies have highlighted both the self-assembly of various lipid formulations and dynamic properties of cellular membranes as they interact with the local environment. Not only have mechanisms of membrane transport and pharmaceutical cargo delivery via liposomes been elucidated, but analytical uses such as immunoassays and biosensors have also been developed. 

At the rudimentary level, most lipids that self assemble into useful shapes are amphipathic, containing both a hydrophilic head group and a hydrophobic lipid tail group. The shapes that are formed are determined by the types of lipids used, which, in turn, provide various options regarding delivery. The cationic head groups appear to be better suited for DNA delivery due to the natural charge attraction between negatively charged phosphate groups and the positively charged head groups. Anionic head groups are perhaps better suited for drug delivery. However, this does not preclude their use as gene delivery vehicles as work with divalent cations has shown. 

One must keep in mind all of the variables that come into play when using different gene delivery vectors. There is no concrete comparison that can easily be made to suggest that one liposomal vector is better than another for all cell types, environments, and applications. While some of the lipids presented above were originally found to yield little-to-no cytotoxicity for a given cell type, the observation does not necessarily hold true when they are applied to different cell types [23–25]. Improvements and adjustments to these formulations are constantly being explored through the addition of different lipids, targeting molecules, or shielding moieties designed to prevent clearance *in vivo*. The identification of the optimal gene delivery vector continues to be an elusive process, and liposomes are but a fraction of all the vehicles that are being examined.

## Figures and Tables

**Figure 3:**
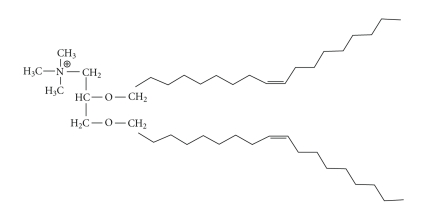
The structure of DOTMA.

**Figure 4:**
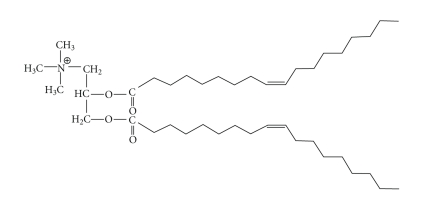
The structure of DOTAP.

**Figure 5:**
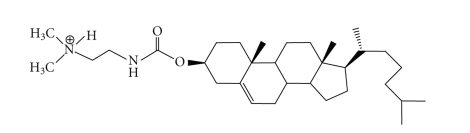
The structure of DC-Chol.

**Figure 6:**
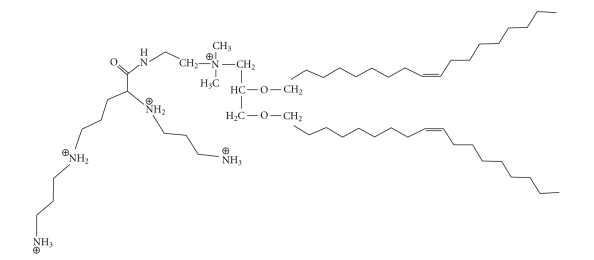
The structure of DOSPA.

**Figure 7:**
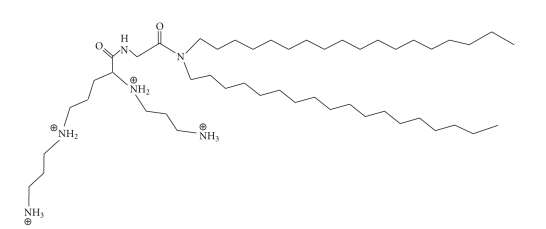
The structure of DOGS.

**Figure 8:**
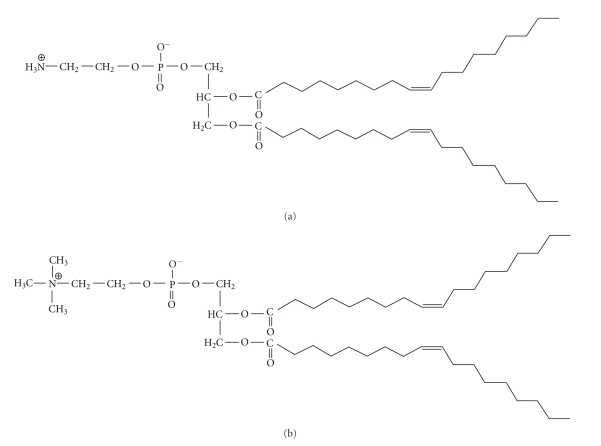
The structures of two neutral lipids. (a) DOPE (b) DOPC.

## References

[1] Bangham AD, Standish MM, Watkins JC (1965). Diffusion of univalent ions across the lamellae of swollen phospholipids. *Journal of Molecular Biology*.

[2] Jesorka A, Orwar O (2008). Liposomes: technologies and analytical applications. *Annual Review of Analytical Chemistry*.

[3] Leduc PR, Wong MS, Ferreira PM (2007). Towards an in vivo biologically inspired nanofactory. *Nature Nanotechnology*.

[4] Montier T, Benvegnu T, Jaffrès P-A, Yaouanc J-J, Lehn P (2008). Progress in cationic lipid-mediated gene transfection: a series of bioinspired lipids as an example. *Current Gene Therapy*.

[5] Tros de Ilarduya C, Sun Y, Düzgüneş N (2010). Gene delivery by lipoplexes and polyplexes. *European Journal of Pharmaceutical Sciences*.

[6] Koltover I, Salditt T, Safinya CR (1999). Phase diagram, stability, and overcharging of lamellar cationic lipid- DNA self-assembled complexes. *Biophysical Journal*.

[7] Gregoriadis G (2006). *Liposome Technology*.

[8] Patil SD, Rhodes DG, Burgess DJ (2004). Anionic liposomal delivery system for DNA transfection. *The AAPS Journal*.

[9] Margineanu D-G (1987). Equilibrium and non-equilibrium approaches in biomembrane thermodynamics. *Archives Internationales de Physiologie et de Biochimie*.

[10] Svetina S, Zeks B (2002). Shape behavior of lipid vesicles as the basis of some cellular processes. *Anatomical Record*.

[11] Shimomura M, Sawadaishi T (2001). Bottom-up strategy of materials fabrication: a new trend in nanotechnology of soft materials. *Current Opinion in Colloid and Interface Science*.

[12] Karmali PP, Chaudhuri A (2007). Cationic liposomes as non-viral carriers of gene medicines: resolved issues, open questions, and future promises. *Medicinal Research Reviews*.

[13] Israelachvili JN (1991). *Intermolecular and Surface Forces*.

[14] Mayhew E, Papajadjopoulos D, Ostro MJ (1983). Therapeutic applications of liposomes. *Liposomes*.

[15] Lasic D (1993). *Liposomes: From Physics to Applications*.

[16] Sternberg B (1994). New structures in complex formation between DNA and cationic liposomes visualized by freeze-fracture electron microscopy. *FEBS Letters*.

[17] Koltover I, Salditt T, Rädler JO, Safinya CR (1998). An inverted hexagonal phase of cationic liposome-DNA complexes related to DNA release and delivery. *Science*.

[18] Rädler JO, Koltover I, Salditt T, Safinya CR (1997). Structure of DNA-cationic liposome complexes: DNA intercalation in multilamellar membranes in distinct interhelical packing regimes. *Science*.

[19] Gustafsson J, Arvidson G, Karlsson G, Almgren M (1995). Complexes between cationic liposomes and DNA visualized by cryo-TEM. *Biochimica et Biophysica Acta*.

[20] Hsu W-L, Chen H-L, Liou W, Lin H-K, Liu W-L (2005). Mesomorphic complexes of DNA with the mixtures of a cationic surfactant and a neutral lipid. *Langmuir*.

[21] Wasungu L, Hoekstra D (2006). Cationic lipids, lipoplexes and intracellular delivery of genes. *Journal of Controlled Release*.

[22] Felgner PL, Gadek TR, Holm M (1987). Lipofection: a highly efficient, lipid-mediated DNA-transfection procedure. *Proceedings of the National Academy of Sciences of the United States of America*.

[23] Leventis R, Silvius JR (1990). Interactions of mammalian cells with lipid dispersions containing novel metabolizable cationic amphiphiles. *Biochimica et Biophysica Acta*.

[24] Gao X, Huang L (1991). A novel cationic liposome reagent for efficient transfection of mammalian cells. *Biochemical and Biophysical Research Communications*.

[25] Behr J-P, Demeneix B, Loeffler J-P, Perez-Mutul J (1989). Efficient gene transfer into mammalian primary endocrine cells with lipopolyamine-coated DNA. *Proceedings of the National Academy of Sciences of the United States of America*.

[26] Farhood H, Serbina N, Huang L (1995). The role of dioleoyl phosphatidylethanolamine in cationic liposome mediated gene transfer. *Biochimica et Biophysica Acta*.

[27] Behr J-P (1994). Gene transfer with synthetic cationic amphiphiles: prospects for gene therapy. *Bioconjugate Chemistry*.

[28] Farhood H, Gao X, Son K (1994). Cationic liposomes for direct gene transfer in therapy of cancer and other diseases. *Annals of the New York Academy of Sciences*.

[29] Kikuchi A, Aoki Y, Sugaya S (1999). Development of novel cationic liposomes for efficient gene transfer into peritoneal disseminated tumor. *Human Gene Therapy*.

[30] Wheeler CJ, Felgner PL, Tsai YJ (1996). A novel cationic lipid greatly enhances plasmid DNA delivery and expression in mouse lung. *Proceedings of the National Academy of Sciences of the United States of America*.

[31] Budker V, Gurevich V, Hagstrom JE, Bortzov F, Wolff JA (1996). pH-sensitive, cationic liposomes: a new synthetic virus-like vector. *Nature Biotechnology*.

[32] Maurer N, Mori A, Palmer L (1999). Lipid-based systems for the intracellular delivery of genetic drugs. *Molecular Membrane Biology*.

[33] Elouahabi A, Ruysschaert J-M (2005). Formation and intracellular trafficking of lipoplexes and polyplexes. *Molecular Therapy*.

[34] Pires P, Simões S, Nir S, Gaspar R, Düzgünes N, Pedroso De Lima MC (1999). Interaction of cationic liposomes and their DNA complexes with monocytic leukemia cells. *Biochimica et Biophysica Acta*.

[35] Ferrari ME, Rusalov D, Enas J, Wheeler CJ (2002). Synergy between cationic lipid and co-lipid determines the macroscopic structure and transfection activity of lipoplexes. *Nucleic Acids Research*.

[36] Remy J-S, Sirlin C, Vierling P, Behr J-P (1994). Gene transfer with a series of lipophilic DNA-binding molecules. *Bioconjugate Chemistry*.

[37] Uchida E, Mizuguchi H, Ishii-Watabe A, Hayakawa T (2002). Comparison of the efficiency and safety of non-viral vector-mediated gene transfer into a wide range of human cells. *Biological and Pharmaceutical Bulletin*.

[38] Malone RW, Felgner PL, Verma IM (1989). Cationic liposome-mediated RNA transfection. *Proceedings of the National Academy of Sciences of the United States of America*.

[39] Ren T, Song YK, Zhang G, Liu D (2000). Structural basis of DOTMA for its high intravenous transfection activity in mouse. *Gene Therapy*.

[40] Felgner JH, Kumar R, Sridhar CN (1994). Enhanced gene delivery and mechanism studies with a novel series of cationic lipid formulations. *Journal of Biological Chemistry*.

[41] Zuidam NJ, Barenholz Y (1998). Electrostatic and structural properties of complexes involving plasmid DNA and cationic lipids commonly used for gene delivery. *Biochimica et Biophysica Acta*.

[42] Zabner J, Fasbender AJ, Moninger T, Poellinger KA, Welsh MJ (1995). Cellular and molecular barriers to gene transfer by a cationic lipid. *Journal of Biological Chemistry*.

[43] Yang J-P, Huang L (1998). Time-dependent maturation of cationic liposome-DNA complex for serum resistance. *Gene Therapy*.

[44] Marchini C, Montani M, Amici A (2009). Structural stability and increase in size rationalize the efficiency of lipoplexes in serum. *Langmuir*.

[45] Zuidam NJ, Barenholz Y (1997). Electrostatic parameters of cationic liposomes commonly used for gene delivery as determined by 4-heptadecyl-7-hydroxycoumarin. *Biochimica et Biophysica Acta*.

[46] Ajmani PS, Hughes JA (1999). 3*β* [N-(NM′, N′-dimethylaminoethane)-carbamoyl] cholesterol (DC-chol)-mediated gene delivery to primary rat neurons: characterization and mechanism. *Neurochemical Research*.

[47] Jain S, Zon G, Sundaralingam M (1989). Base only binding of spermine in the deep groove of the A-DNA octamer d(GTGTACAC). *Biochemistry*.

[48] Boukhnikachvili T, Aguerre-Chariol O, Airiau M, Lesieur S, Ollivon M, Vacus J (1997). Structure of in-serum transfecting DNA-cationic lipid complexes. *FEBS Letters*.

[49] Metselaar JM, Bruin P, De Boer LWT (2003). A novel family of L-amino acid-based biodegradable polymer-lipid conjugates for the development of long-circulating liposomes with effective drug-targeting capacity. *Bioconjugate Chemistry*.

[50] Immordino ML, Dosio F, Cattel L (2006). Stealth liposomes: review of the basic science, rationale, and clinical applications, existing and potential. *International Journal of Nanomedicine*.

[51] Kim J-K, Choi S-H, Kim C-O, Park J-S, Ahn W-S, Kim C-K (2003). Enhancement of polyethylene glycol (PEG)-modified cationic liposome-mediated gene deliveries: effects on serum stability and transfection efficiency. *Journal of Pharmacy and Pharmacology*.

[52] Needham D, McIntosh TJ, Lasic DD (1992). Repulsive interactions and mechanical stability of polymer-grafted lipid membranes. *Biochimica et Biophysica Acta*.

[53] DeCastro M, Saijoh Y, Schoenwolf GC (2006). Optimized cationic lipid-based gene delivery reagents for use in developing vertebrate embryos. *Developmental Dynamics*.

[54] Shi F, Wasungu L, Nomden A (2002). Interference of poly(ethylene glycol)-lipid analogues with cationic-lipid-mediated delivery of oligonucleotides; role of lipid exchangeability and non-lamellar transitions. *Biochemical Journal*.

[55] Ishida O, Maruyama K, Tanahashi H (2001). Liposomes bearing polyethyleneglycol-coupled transferrin with intracellular targeting property to the solid tumors in vivo. *Pharmaceutical Research*.

[56] Mukherjee A, Prasad TK, Rao NM, Banerjee R (2005). Haloperidol-associated stealth liposomes: a potent carrier for delivering genes to human breast cancer cells. *Journal of Biological Chemistry*.

[57] Simões S, Slepushkin V, Caspar R, Pedroso de Lima MC, Düzgüneş N (1998). Gene delivery by negatively charged ternary complexes of DNA, cationic liposomes and transferrin or fusigenic peptides. *Gene Therapy*.

[58] Chesnoy S, Huang L (2000). Structure and function of lipid-DNA complexes for gene delivery. *Annual Review of Biophysics and Biomolecular Structure*.

[59] Zuhorn IS, Bakowsky U, Polushkin E (2005). Nonbilayer phase of lipoplex-membrane mixture determines endosomal escape of genetic cargo and transfection efficiency. *Molecular Therapy*.

[60] Bennett MJ, Nantz MH, Balasubramaniam RP, Gruenert DC, Malone RW (1995). Cholesterol enhances cationic liposome-mediated DNA transfection of human respiratory epithelial cells. *Bioscience Reports*.

[61] Hong K, Zheng W, Baker A, Papahadjopoulos D (1997). Stabilization of cationic liposome-plasmid DNA complexes by polyamines and poly(ethylene glycol)-phospholipid conjugates for efficient in vivo gene delivery. *FEBS Letters*.

[62] Kawakami S, Yamashita F, Nishikawa M, Takakura Y, Hashida M (1998). Asialoglycoprotein receptor-mediated gene transfer using novel galactosylated cationic liposomes. *Biochemical and Biophysical Research Communications*.

[63] Marshall J, Yew NS, Eastman SJ, Jiang C, Scheule RK, Cheng SH, Huang L, Hung M-C, Wagner E (1999). Cationic lipid-mediated gene delivery to the airways. *Nonviral Vectors for Gene Therapy*.

[64] Lappalainen K, Jaaskelainen I, Syrjanen K, Urtti A, Syrjanen S (1994). Comparison of cell proliferation and toxicity assays using two cationic liposomes. *Pharmaceutical Research*.

[65] Dokka S, Toledo D, Shi X, Castranova V, Rojanasakul Y (2000). Oxygen radical-mediated pulmonary toxicity induced by some cationic liposomes. *Pharmaceutical Research*.

[66] Srinivasan C, Burgess DJ (2009). Optimization and characterization of anionic lipoplexes for gene delivery. *Journal of Controlled Release*.

[67] Roerdink F, Wassef NM, Richardson EC, Alving CR (1983). Effects of negatively charged lipids on phagocytosis of liposomes opsonized by complement. *Biochimica et Biophysica Acta*.

[68] Lakkaraju A, Dubinsky JM, Low WC, Rahman Y-E (2001). Neurons are protected from excitotoxic death by p53 antisense oligonucleotides delivered in anionic liposomes. *Journal of Biological Chemistry*.

[69] Patil SD, Rhodes DG, Burgess DJ (2005). Biophysical characterization of anionic lipoplexes. *Biochimica et Biophysica Acta*.

[70] Lasic DD, Templeton NS (1996). Liposomes in gene therapy. *Advanced Drug Delivery Reviews*.

[71] Kulkarni VI, Shenoy VS, Dodiya SS, Rajyaguru TH, Murthy RR (2006). Role of calcium in gene delivery. *Expert Opinion on Drug Delivery*.

[72] Rejman J, Oberle V, Zuhorn IS, Hoekstra D (2004). Size-dependent internalization of particles via the pathways of clathrin-and caveolae-mediated endocytosis. *Biochemical Journal*.

[73] Koynova R, MacDonald RC (2007). Natural lipid extracts and biomembrane-mimicking lipid compositions are disposed to form nonlamellar phases, and they release DNA from lipoplexes most efficiently. *Biochimica et Biophysica Acta*.

[74] Scherphof G, Roerdink F, Waite M, Parks J (1978). Disintegration of phosphatidylcholine liposomes in plasma as a result of interaction with high-density lipoproteins. *Biochimica et Biophysica Acta*.

[75] Comiskey SJ, Heath TD (1990). Serum-induced leakage of negatively charged liposomes at nanomolar lipid concentrations. *Biochemistry*.

